# Oncogenic Role of Exosomal Circular and Long Noncoding RNAs in Gastrointestinal Cancers

**DOI:** 10.3390/ijms23020930

**Published:** 2022-01-15

**Authors:** Ba Da Yun, Ye Ji Choi, Seung Wan Son, Gabriel Adelman Cipolla, Fernanda Costa Brandão Berti, Danielle Malheiros, Tae-Jin Oh, Hyo Jeong Kuh, Soo Young Choi, Jong Kook Park

**Affiliations:** 1 Department of Biomedical Science and Research, Institute for Bioscience & Biotechnology, Hallym University, Chunchon 24252, Korea; dbsbada@naver.com (B.D.Y.); cdw4572@naver.com (Y.J.C.); miyanae@naver.com (S.W.S.); sychoi@hallym.ac.kr (S.Y.C.); 2 Postgraduate Program in Genetics, Department of Genetics, Federal University of Paraná, Curitiba 81531-990, Brazil; gabriel.cipolla@ufpr.br (G.A.C.); fernanda.berti@ufpr.br (F.C.B.B.); dani_malheiros@ufpr.br (D.M.); 3 Department of Pharmaceutical Engineering and Biotechnology, SunMoon University, 70 Sunmoon-ro 221, Tangjeong-myeon, Asan-si 31460, Korea; tjoh3782@sunmoon.ac.kr; 4 Genome-Based BioIT Convergence Institute, 70 Sunmoon-ro 221, Tangjeong-myeon, Asan-si 31460, Korea; 5 Department of Medical Life Sciences, College of Medicine, The Catholic University of Korea, Seoul 06591, Korea; hkuh@catholic.ac.kr

**Keywords:** exosome, circular RNA, long noncoding RNA, gastrointestinal cancer

## Abstract

Circular RNAs (circRNAs) and long noncoding RNAs (lncRNAs) are differentially expressed in gastrointestinal cancers. These noncoding RNAs (ncRNAs) regulate a variety of cellular activities by physically interacting with microRNAs and proteins and altering their activity. It has also been suggested that exosomes encapsulate circRNAs and lncRNAs in cancer cells. Exosomes are then discharged into the extracellular environment, where they are taken up by other cells. As a result, exosomal ncRNA cargo is critical for cell–cell communication within the cancer microenvironment. Exosomal ncRNAs can regulate a range of events, such as angiogenesis, metastasis, immune evasion, drug resistance, and epithelial-to-mesenchymal transition. To set the groundwork for developing novel therapeutic strategies against gastrointestinal malignancies, a thorough understanding of circRNAs and lncRNAs is required. In this review, we discuss the function and intrinsic features of oncogenic circRNAs and lncRNAs that are enriched within exosomes.

## 1. Introduction

Solid cancers of the gastrointestinal system, such as colorectal cancer, esophageal cancer, gastric cancer, hepatocellular carcinoma, and pancreatic cancer, are among the most frequent types of malignancies. According to the GLOBOCAN database, gastrointestinal cancers are responsible for 26% of cancer incidence and 35% of cancer-related mortality worldwide [1]. Due to the lack of curative options, little progress has been made in the treatment of patients with gastrointestinal malignancies. Several classes of drugs, such as sorafenib, paclitaxel, and cetuximab, have been approved for use. However, the overall survival of patients and their quality of life have not improved significantly [2,3]. In order to develop more effective and appropriate treatment strategies, it is critical to identify novel target molecules associated with gastrointestinal cancers.

In solid cancers, the microenvironment is heterogeneous and composed of cellular and noncellular components, including cancer-associated fibroblasts (CAFs), immune cells, and the extracellular matrix [4,5]. Hypoxia, one of the characteristics of the cancer microenvironment, is caused by factors such as uncontrolled cancer growth and leads to metabolic reprogramming [6]. Reciprocal communication among cells enables them to share diverse cellular factors within the microenvironment. Exosomes are a class of extracellular vesicles that act as key players in this event, affecting cancer aggressiveness, anti-cancer immune responses, and treatment efficacy [7,8]. For instance, annexin A1 (*ANXA1*) proteins are cargo molecules in cancer cell-derived exosomes and activate epithelial–mesenchymal transition (EMT) in exosome-receiving cancer cells [9]. Another example is the potential of exosomal protein cargo, macrophage migration inhibitory factor (*MIF*), to reshape the phenotype of drug-sensitive cancer cells into temozolomide-resistant cells by positively modulating phosphoinositide 3-kinase (PI3K)/AKT signaling. The pharmacological inhibition of MIF prominently enhances temozolomide efficacy in vivo [10]. In addition, gemcitabine resistance is exacerbated by CAF-derived exosomes that harbor snail family transcriptional repressor 1 (*SNAI1*) messenger RNA (mRNA). Anti-pancreatic cancer effects of gemcitabine can be improved by pharmacological interruption of exosome secretion using GW4869 [11].

Circular RNAs (circRNAs) and long noncoding RNAs (lncRNAs) are aberrantly expressed in cancer and impinge on the activity of target molecules, including microRNA (miRNA) and proteins, therefore controlling cancer progression, therapeutic resistance, etc. [12,13,14,15,16]. One of the known functions of both circRNAs and lncRNAs is to act as competitive endogenous RNAs by sequestering miRNAs. For example, recent studies demonstrated that circ_ZFPM2 and lncRNA-ADPGK-AS1 can promote EMT by molecularly sponging miR-205-5p [17,18]. Furthermore, circRNAs interact with RNA-binding proteins to control gene transcription [19,20]. LncRNAs have been found to regulate a variety of events, including RNA splicing and protein degradation [21].

These noncoding RNAs (ncRNAs) can be incorporated and differentially loaded into exosomes. For instance, circ_EPB41L2 quantity is low in exosomes secreted by colorectal cancer cell lines. Treatment of cancer cells with exosomes acquired from circ_EPB41L2-overexpressing cells retards cell proliferation, survival, migration, and invasion by negatively modulating the effect of miR-21-5p and miR-942-5p on phosphatase and tensin homolog (*PTEN*) mRNA [22]. This review focuses on exosome-enriched circRNAs and lncRNAs to meticulously understand their oncogenic role in gastrointestinal cancers. We also present an overview of the relevant features of circRNAs and lncRNAs.

## 2. Exosomal CircRNAs

### 2.1. Colorectal Cancer

#### 2.1.1. Circ_0000338

**Figure 1:**
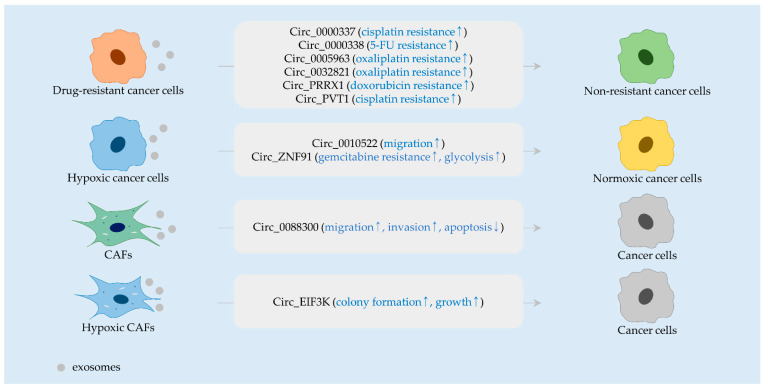
Exosomal circRNAs secreted from drug-resistant cancer cells, hypoxic cancer cells, CAFs, and hypoxic CAFs. CircRNAs (black), and their biological functions (blue) are indicated in rounded rectangles. Arrows indicate the upregulation (↑) and downregulation (↓).

It was demonstrated that drug-sensitive parental cells cocultured with FOLFOX-resistant cells become desensitized to 5-fluorouracil (5-FU), implying that exosome-transferred molecules contribute to chemoresistance. Profiling of circRNAs revealed that circ_0000338 is one of the upregulated circRNAs in exosomes secreted from FOLFOX-resistant cells [23]. Another study further showed that circ_0000338 levels are upregulated in cancer tissues and cell lines and that the silencing of circ_0000338 sensitizes resistant cells to 5-FU via enhancing apoptotic cell death [24]. Treatment of cells with exosomes harboring circ_0000338 reduces the efficacy of 5-FU in vitro and in vivo. Mechanistically, it was identified that circ_0000338 directly interacts with and inactivates miR-217 and miR-485-3p [24] (Figure 1 and Table 1). It is known that miR-217 and miR-485-3p target mitogen-activated protein kinase 1 (*MAPK1*) and maternal embryonic leucine zipper kinase (*MELK*), respectively [25,26]. Since MAPK1 and MELK can promote 5-FU resistance [27,28], circ_0000338 may regulate drug sensitivity, partly via modulating the level of both MAPK1 and MELK.

#### 2.1.2. Circ_0005963

Glycolysis has been found to be associated with therapeutic resistance toward several classes of anti-cancer drugs. Lactate, a glycolytic metabolite, can induce drug resistance by creating an acidic tumor microenvironment [29]. In addition, drug resistance can also be due to a glycolysis-induced upregulation of drug efflux pumps. For example, pyruvate kinase M2 type (*PKM2*), a glycolytic enzyme, facilitates oxaliplatin resistance via upregulating ATP-binding cassette subfamily B member 1 (*ABCB1*, also called *P-glycoprotein 1*) [30]. Recently, it was reported that circ_0005963 is enriched in exosomes from oxaliplatin-resistant cells and the serum of an oxaliplatin-resistant patient [31]. In this study, circ_0005963 was observed to be delivered from oxaliplatin-resistant cells to sensitive cells via exosomes. In recipient cells, circ_0005963 increases PKM2 expression via inactivating miR-122, ultimately diminishing the anti-colorectal cancer effect of oxaliplatin. Knockdown of circ_0005963 augments oxaliplatin-induced apoptotic cell death in vitro and inhibits cancer growth in vivo [31] (Figure 1 and Table 1).

**Table 1:** Exosomal circRNAs in gastrointestinal cancers (in alphanumerical order).

Cargo Molecule	Expression	Source of Exosome	Type of Cancer	Target Molecule	Clinical Relevance	Ref.
Circ_0000337	Up	Cisplatin-resistant EC9706 and KYSE30 cells	Esophageal cancer	miR-377-3p	-	[32]
Circ_0000338	Up	FOLFOX-resistant HCT116 cells	Colorectal cancer	-	-	[23]
	Up	5-FU-resistant SW480 and HCT116 cells	Colorectal cancer	miR-217 and miR-485-3p	5-FU resistance is associated with high levels of circ_0000338	[24]
Circ_0005963	Up	Serum from oxaliplatin-resistant patients. Oxaliplatin-resistant SW480 cells	Colorectal cancer	miR-122	Highly expressed in oxaliplatin-resistant patients	[31]
Circ_0010522	Up	SW480 and HCT116 cells exposed to a hypoxic condition (1% O_2_)	Colorectal cancer	miR-133a-3p	Positively associated with the stages of cancer	[33]
Circ_0032821	Up	Oxaliplatin-resistant HGC-27 and AGS cells	Gastric cancer	miR-515-5p	-	[34]
Circ_0044366	Up	Patient plasma. SGC-7901 and MGC-803 cells	Gastric cancer	miR-29a	-	[35]
Circ_0061395	Up	Patient serum	Hepatocellular carcinoma	miR-877-5p	-	[36]
Circ_0067835	Up	Serum from radiotherapy-treated patients. SW620 and HCT116 cells	Colorectal cancer	miR-296-5p	A diagnostic biomarker candidate	[37]
Circ_0088300	Up	Patient plasma. CAFs	Gastric cancer	miR-1305	Negative correlation between circ_0088300 expression and survival probability	[38]
Circ_EIF3K	Up	Hypoxic CAFs	Colorectal cancer	miR-214	Poor overall survival of patients with high circ_EIF3K levels	[39]
Circ_FBLIM1	Up	Patient serum	Hepatocellular carcinoma	miR-338-3p	-	[40]
Circ_IARS	Up	Patient plasma. Hs766T and Hs766T-L2 cells	Pancreatic cancer	miR-122	Associated with vascular invasion; liver metastasis; and advanced tumor, node, and metastasis (TNM) stage	[41]
Circ_IFT80	Up	Patient serum. SW480 and SW620 cells	Colorectal cancer	miR-296-5p	-	[42]
Circ_MMP2	Up	LM3 and 97H cells	Hepatocellular carcinoma	miR-136-5p	Low survival rate of patients with high circ_MMP2 expression	[43]
Circ_NEK9	Up	Patient plasma	Gastric cancer	miR-409-3p	Associated with TNM stage	[44]
Circ_NHSL1	Up	Patient serum. HGC-27 and AGS cells	Gastric cancer	miR-149-5p	Associated with lymphatic metastasis and TNM stage	[45]
Circ_PACRGL	Up	HCT116 and SW480 cells	Colorectal cancer	miR-142-3p and miR-506-3p	-	[46]
Circ_PDE8A	Up	Patient plasma. Hs766T-L2 cells	Pancreatic cancer	miR-338-3p	Associated with lymphatic invasion and TNM stage	[47]
Circ_PRRX1	Up	Doxorubicin-resistant HGC-27 and AGS cells	Gastric cancer	miR-3064-5p	Associated with doxorubicin resistance	[48]
Circ_PVT1	Up	Serum from cisplatin-resistant patients. Cisplatin-resistant HGC-27 and AGS cells	Gastric cancer	miR-30a-5p	Associated with lymph node metastasis and tumor size	[49]
Circ_SHKBP1	Up	Patient serum. BGC823 and HGC27 cells	Gastric cancer	miR-582-3p	Positively associated with poor prognosis, vascular invasion, and TNM stage	[50]
Circ_TMEM45A	Up	Patient serum	Hepatocellular carcinoma	miR-665	Correlated with poor prognosis and clinicopathological parameters such as TNM stage	[51]
Circ_UHRF1	Up	HCCLM3 and SMMC-7721 cells	Hepatocellular carcinoma	miR-449c-5p	Poor clinical prognosis of patients with high circ_UHRF1 expression	[52]
Circ_ZNF652	Up	Patient serum. SNU-387 and Huh7 cells	Hepatocellular carcinoma	miR-29a-3p	-	[53]
Circ_ZNF91	Up	Hypoxic BxPC-3 cells	Pancreatic cancer	miR-23b-3p	Worse overall survival of patients with high circ_ZNF91 expression	[54]

#### 2.1.3. Circ_0010522 and Circ_EIF3K

Guanine nucleotide exchange factor H1 (*GEF-H1*) serves as an activator of Ras homolog family member A (*RhoA*), promoting cell motility, invasion, and metastasis [55]. A recent study showed that circ_0010522 is present in exosomes secreted from hypoxic cancer cells [33]. Migratory capacities of normoxic cells are increased by treatment with hypoxic exosomes, suggesting a pro-migratory activity of circ_0010522. It was further shown that circ_0010522 absorbs miR-133a-3p, thereby elevating GEF-H1 and RhoA levels [33] (Figure 1 and Table 1).

Secretion of exosomes from CAFs is stimulated by hypoxic conditions [39]. Further investigation demonstrated that these exosomes carry circ_EIF3K, which sponges miR-214, which targets programmed cell death 1 ligand 1 (*PD-L1*). Colony formation of cancer cells is attenuated by culturing with conditioned media derived from circ_EIF3K-silenced CAFs. The growth of cancer is also impaired in xenografts treated with exosomes from circ_EIF3K-silenced CAFs. These results denote the role of exosomal circ_EIF3K in cancer progression [39] (Figure 1 and Table 1).

#### 2.1.4. Circ_0067835 and Circ_IFT80

**Figure 2:**
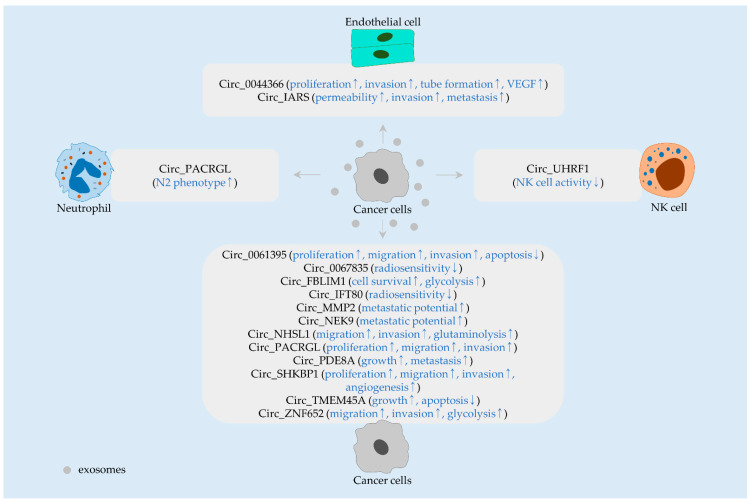
Exosomal circRNAs released from cancer cells and their effects in recipient cells. CircRNAs (black), and their biological functions (blue) are denoted in rounded rectangles. Arrows indicate the upregulation (↑) and downregulation (↓).

Circ_0067835 is abundant in blood-derived exosomes from patients who received radiotherapy, suggesting an involvement of this circRNA in radiosensitivity modulation. Indeed, it was noticed that radiotherapy-induced apoptosis is increased in cancer cells treated with exosomes derived from circ_0067835-silenced cells [37]. Furthermore, exosomal circ_IFT80 desensitizes recipient cells to radiation [42]. In their studies, circ_0067835 and circ_IFT80 were found to positively regulate the level of insulin-like growth factor 1 receptor (*IGF1R*) and musashi-1 (*MSI1*), respectively, by sponging miR-296-5p [37,42] (Figure 2 and Table 1). IGF1R and MSI1 have been investigated to reduce radiosensitivity by activating DNA damage repair [56,57].

#### 2.1.5. Circ_PACRGL

Microarray analysis showed that circ_PACRGL is copiously detected in exosomes released from cancer cells [46]. Functional investigation exhibited that exosomal circ_PACRGL stimulates cell proliferation, migration, and invasion by sponging miR-142-3p and miR-506-3p, both of which directly regulate transforming growth factor beta 1 (*TGFB1*). In addition, exosomal circ_PACRGL stimulates neutrophil polarization towards a pro-tumorigenic N2 phenotype [46], suggesting that circ_PACRGL is a promising target for cancer treatment (Figure 2 and Table 1).

### 2.2. Esophageal Cancer

Circ_0000337

Circ_0000337 has been discovered to be an oncogenic factor. In osteosarcoma, circ_0000337 augments proliferation and migration by elevating BTB domain and CNC homolog 1 (*BACH1*) levels [58]. Besides this, circ_0000337 upmodulates methionine adenosyltransferase 2A (*MAT2A*) expression, thus accelerating migration and invasion in glioblastoma cells [59]. Circ_0000337 is highly expressed in esophageal cancer tissues and enhances the metastatic potential of cancer cells [60]. Moreover, it was found that circ_0000337 is highly expressed in cisplatin-resistant esophageal cancer cells and abundant in their secreted exosomes [32]. Non-resistant parental cells become less sensitive to cisplatin following exposure to exosomes from resistant cells in vitro and in vivo. Circ_0000337 can aggravate cisplatin resistance due to its interaction with miR-377-3p, which targets Janus kinase 2 (*JAK2*) [32] (Figure 1 and Table 1). However, miR-377-3p was shown to increase glycogen synthase kinase 3 beta (*GSK3B*) expression and activate nuclear factor kappa B (NF-κB) signaling [61]. Therefore, circ_0000337 knockdown may unexpectedly activate GSK3B/NF-κB signaling.

### 2.3. Gastric Cancer

#### 2.3.1. Circ_0032821, Circ_PRRX1, and Circ_PVT1

It has been evaluated that miR-515-5p attenuates chemoresistance. The efficacy of vincristine and carboplatin is improved by miR-515-5p in retinoblastoma [62]. Further, miR-515-5p can negatively control interleukin 25 (IL25) levels and sensitize resistant cells to cisplatin as well as to 5-FU in nasopharyngeal cancer [63]. In gastric cancer, circ_0032821 competitively interacts with miR-515-5p to control SRY-box transcription factor 9 (*SOX9*) expression [34]. Owing to such an ability of circ_0032821, oxaliplatin resistance can be provoked in drug-sensitive cells after exposure to exosomal circ_0032821 derived from oxaliplatin-resistant cells [34] (Figure 1 and Table 1).

Protein tyrosine phosphatase non-receptor type 14 (*PTPN14*) functions as an oncogenic factor by advancing the proliferation and migration of gastric cancer cells via triggering Hippo signaling [64]. Additionally, PTPN14 overexpression can upregulate and downregulate the level of vimentin and caspase-3, respectively, in gastric cancer [65]. Analyses of the relationship between circ_PRRX1 and PTPN14 showed that circ_PRRX1 is able to positively control PTPN14 levels via sequestering miR-3064-5p, consequently intensifying doxorubicin resistance in gastric cancer [48]. Circ_PRRX1 is bountiful in exosomes released from doxorubicin-resistant cells. The effect of doxorubicin on proliferation, migration, and invasion is attenuated in exosome-receiving cancer cells [48] (Figure 1 and Table 1).

Circ_PVT1 is involved in the regulation of a variety of cellular events. Circ_PVT1 overexpression increases the invasive capacity of esophageal and gallbladder cancer cells [66,67]. In addition, circ_PVT1 makes lung cancer cells less responsive to Pemetrexed and cisplatin by elevating the expression of ABCC1 (also known as multidrug resistance-associated protein 1, *MRP1*) [68]. In gastric cancer, circ_PVT1 is profusely present in exosomes from cisplatin-resistant cancer cells [49]. In their study, circ_PVT1 was observed to negatively control miR-30a-5p, which targets Yes-associated protein 1 (*YAP1*). The silencing of circ_PVT1 re-sensitizes resistant cancer cells to cisplatin by augmenting apoptotic cell death and suppressing autophagy [49]. These findings suggest that exosomal circ_PVT1 is responsible for cisplatin resistance in recipient cells, at least partly via ABCC1 and YAP1 (Figure 1 and Table 1).

#### 2.3.2. Circ_0044366 and Circ_SHKBP1

Exosomal circRNAs play a part in angiogenesis regulation. Treatment with cancer cell-derived exosomal circ_0044366 significantly enhances the proliferation, invasion, and tube formation of human umbilical vein endothelial cells (HUVECs) in vitro. Besides this, exosomal circ_0044366 promotes cancer growth and angiogenesis in vivo. Mechanistically, exosomal circ_0044366 leads to an increment of vascular endothelial growth factor (VEGF) levels via inactivating miR-29a in endothelial cells [35] (Figure 2 and Table 1).

Circ_SHKBP1 levels are higher in cancer tissues and exosomes derived from patient serum and cancer cell lines [50]. Exosomal circ_SHKBP1 facilitates cell proliferation, migration, and invasion. Furthermore, exosomal circ_SHKBP1 increases Hu-antigen R (*HUR*) levels via inactivating miR-582-3p, resulting in an increment of VEGF expression in cancer cells in vitro. Cancer growth and angiogenesis are activated by circ_SHKBP1 in vivo [50] (Figure 2 and Table 1). Other evidence showed that circ_SHKBP1 supports survival and tube formation of endothelial cells [69]. Therefore, these results suggest that circ_SHKBP1 can also be transferred to endothelial cells via exosomes, which, in turn, stimulate angiogenic events.

#### 2.3.3. Circ_0088300

Once activated, JAK/signal transducer and activator of transcription (STAT) signaling promotes the expression of target genes involved in diverse cellular events, such as cell survival, migration, invasion, and therapeutic resistance [70]. Because JAK/STAT signaling is abnormally activated in gastric cancer, inhibiting this pathway has been proposed as a therapeutic choice [71]. Lately, circ_0088300 was identified as an exosomal circRNA from CAFs. Gastric cancer cells treated with conditioned media from CAFs express high circ_0088300 levels [38]. Circ_0088300 inactivates miR-1305, a negative regulator of JAK1 and STAT1. The overexpression of circ_0088300 reduces the level of pro-apoptotic factors such as BCL2-associated X protein (*BAX*) in cancer cells and enhances migration and invasion [38]. These observations reveal circ_0088300 as a mediator of CAF-induced cancer progression (Figure 1 and Table 1).

#### 2.3.4. Circ_NEK9 and Circ_NHSL1

Both miR-149-5p and miR-409-3p perform a tumor-suppressive function. By targeting BCL2 and cell division cycle 42 (*CDC42*), miR-149-5p promotes apoptotic cell death, represses cell proliferation, and alleviates doxorubicin resistance in neuroblastomas [72]. In gastric cancer, miR-149-5p has an anti-metastatic property by suppressing forkhead box M1 (*FOXM1*) expression [73]. In addition, miR-409-3p is downregulated and capable of suppressing proliferation, invasion, survival, and metastasis by regulating the level of radixin and PHD finger protein 10 (*PHF10*) in gastric cancer [74,75].

Further functional studies showed that circ_NEK9 and circ_NHSL1 inactivate miR-409-3p and miR-149-5p, respectively. By suppressing such miRNAs, circ_NEK9 and circ_NHSL1 elevate the expression of microtubule-associated protein 7 (*MAP7*) and tyrosine 3-monooxygenase/tryptophan 5-monooxygenase activation protein zeta (*YWHAZ*), respectively, in cancer cells [44,45]. Circ_NEK9 is upregulated in gastric cancer cells and abundant in exosomes from patient plasma. These results suggest that exosomal circ_NEK9 is originated from cancer cells rather than other cellular components within the tumor microenvironment. The metastatic ability of cancer cells is intensified by plasma exosomes, connoting the contribution of exosomal circ_NEK9 to gastric cancer progression by raising MAP7 levels [44]. Circ_NHSL1 is enriched in exosomes from cancer cells; therefore, it is feasible that exosomal circ_NHSL1 facilitates migration, invasion, and glutaminolysis in recipient cancer cells by upregulating YWHAZ expression [45] (Figure 2 and Table 1).

### 2.4. Hepatocellular Carcinoma

#### 2.4.1. Circ_0061395

Circ_0061395 (also called circ_BACH1) is notably overexpressed in hepatocellular carcinomas. It was also observed that circ_0061395 can be incorporated into exosomes [36]. Functional analyses indicated that circ_0061395 inactivates miR-877-5p and contributes to cancer progression by positively modulating the expression of phosphoinositide-3-kinase regulatory subunit 3 (*PIK3R3*) [36] (Figure 2 and Table 1). Other studies demonstrated that circ_0061395 can promote cell proliferation and EMT via upregulating SERBP1 and translocating HUR to the cytoplasm [76,77]. Since EMT is one of the causes of therapeutic resistance [78,79], both EMT and drug resistance may be aggravated by the exosomal transport of circ_0061395 between hepatocellular carcinoma cells. Additionally, circ_0061395 is overexpressed in colorectal cancer; supports cell proliferation, migration, and invasion; and restricts apoptotic cell death [80]. Thus, it is worth investigating the existence and amount of circ_0061395 in exosomes released from colorectal cancer cells.

#### 2.4.2. Circ_FBLIM1

It was denoted that circ_FBLIM1 is overexpressed in hepatocellular carcinoma tissues and cell lines [81]. Additionally, circ_FBLIM1 is plenteous in serum exosomes from patients [40], suggesting that exosomal circ_FBLIM1 can be originated from cancer cells. Treatment of hepatocellular carcinoma cells with serum exosomes leads to an upregulation of circ_FBLIM1 levels [40]. In their study, this circRNA was validated to deactivate miR-338-3p, thus triggering cancer progression and glycolysis by increasing the level of low-density lipoprotein receptor-related protein 6 (*LRP6*), which acts as a coreceptor of WNT ligands and activates WNT/β-catenin signaling [40]. Hence, circ_FBLIM1 may be transported between cancer cells to regulate the miR-338-3p/LRP6/WNT/β-catenin axis (Figure 2 and Table 1).

Another study showed that knockdown of circ_FBLIM1 hampers cell proliferation and invasion while promoting apoptotic cell death by augmenting miR-346 levels [81]. Even though circ_FBLIM1 can interact with miR-346, this miRNA has been reported to act as an oncogenic miRNA in various cancer types, including hepatocellular carcinoma [82,83,84]. Such findings point to the possibility that suppression of oncogenic circRNAs compensatorily activates a set of oncogenic miRNAs and their relevant signaling, eventually contributing to the recovery of damaged cancer cells.

#### 2.4.3. Circ_MMP2

In lung cancer, circ_MMP2 (also known as circ_0039411) can bind to insulin-like growth factor 2 mRNA binding protein 3 (*IGF2BP3*), thus expediting cell proliferation and EMT by stabilizing FOXM1 transcripts [85]. Besides this, circ_MMP2 was determined to advance the progression of papillary thyroid cancer by post-transcriptionally upregulating ABCA9 and metastasis-associated 1 (*MTA1*) [86]. Such an oncogenic role of circ_MMP2 was also reported in hepatocellular carcinomas [43]. Circ_MMP2 is plentiful in cancer cell-derived exosomes and can be transported into other cancer cells. Circ_MMP2 is involved in an increase of the metastatic potential of exosome-receiving cancer cells via modulating the miR-136-5p/matrix metallopeptidase 2 (*MMP2*) axis [43] (Figure 2 and Table 1).

#### 2.4.4. Circ_TMEM45A

Functional investigations indicated that this circRNA blocks apoptotic cell death and promotes in vivo cancer growth via regulating the miR-665/insulin growth factor 2 (*IGF2*) axis [51]. Circ_TMEM45A is one of the highly expressed circRNAs in hepatocellular carcinoma and is bountifully present in serum exosomes as well [51]. Therefore, sharing of circ_TMEM45A between cancer cells may contribute to IGF2 upregulation (Figure 2 and Table 1).

Inconsistent with the IGF2-targeting property of miR-665, other studies showed that miR-665 is overexpressed in hepatocellular carcinomas and prompts cell proliferation as well as metastasis [87,88]. Thus, circ_TMEM45A may interact with other potential target proteins and unidentified miRNAs, rather than miR-665, to fulfill its oncogenic role in hepatocellular carcinomas.

#### 2.4.5. Circ_UHRF1

A recent study showed that circ_UHRF1 accelerates cell proliferation and EMT by elevating MYC proto-oncogene (*MYC*) expression in oral squamous cell carcinomas [89]. Further, circ_UHRF1 is upregulated in hepatocellular carcinoma tissues, and exosomal circ_UHRF1 is largely released from cancer cells [52]. Exosomal circ_UHRF1 was revealed to impair the function of natural killer (NK) cells by sequestering miR-449c-5p that directly targets hepatitis A virus cellular receptor 2 (*HAVCR2*, also named *TIM3*), ultimately diminishing the anti-cancer effects of nivolumab, anti-programmed cell death 1 (*PD1*) antibodies [52] (Figure 2 and Table 1).

It has been suggested that HAVCR2 is a molecular marker of activated NK cells and that NK cell-mediated cytotoxicity can be inhibited by anti-HAVCR2 antibodies [90]. By contrast, another study showed that NK cell-mediated cytotoxicity is improved by an HAVCR2 blockade [91]. Such a double-edged function of HAVCR2 is thought to be dependent on the type of HAVCR2 ligands [90,91]. Therefore, it is necessary to reveal the precise function of HAVCR2 and its contribution to the oncogenic effect of circ_UHRF1.

#### 2.4.6. Circ_ZNF652

It has been proven that miR-203 and miR-502-5p are tumor-suppressive miRNAs in gastrointestinal cancers. For instance, miR-203 negatively modulates the level of eukaryotic translation initiation factor 5A2 (*EIF5A2*) in colorectal cancer, restraining cancer progression [92]. In the case of miR-502-5p, this miRNA can suppress cytoprotective autophagy in colorectal cancer [93]. In addition to this, miR-502-5p was recently explored to target Sp1 transcription factor (*SP1*), thus impeding migration and invasion of gastric cancer cells [94].

In hepatocellular carcinoma, miR-203 and miR-502-5p commonly target SNAI1, an EMT-promoting factor, and they are sponged by circ_ZNF652; therefore, lung metastasis is provoked by circ_ZNF652 [95]. Furthermore, circ_ZNF652 is an exosomal circRNA in hepatocellular carcinomas [53]. Circ_ZNF652 is significantly detected in exosomes derived from patient serum and cancer cells. The silencing of circ_ZNF652 suppresses migration, invasion, and glycolysis in exosome-receiving cells. The mechanism underlying circ_ZNF652-mediated cellular events involves the regulation of the miR-29a-3p/guanylyl cyclase domain containing 1 (*GUCD1*) axis [53] (Figure 2 and Table 1). Interestingly, circ_ZNF652 increases PTEN expression, thus positively regulating lipopolysaccharide-induced apoptosis in chondrocytes [96], indicating that the function of circ_ZNF652 can be distinct, depending on the cellular context.

### 2.5. Pancreatic Cancer

#### 2.5.1. Circ_IARS

Endothelial tight junctions act as permeability barriers and restrict cancer metastasis. Transendothelial permeability of cancer cells is induced by the downregulation of Zona occludens-1 (*ZO-1*), a master regulator of tight junctions [97]. It has been noticed that endothelial tight junctions are disrupted by cancer-secreted factors, such as MMP2, MMP9, and miR-105, inducing vascular permeability and metastasis [98]. Besides, it was demonstrated that circ_IARS is overexpressed in pancreatic cancer tissues and especially abundant in plasma exosomes from patients with metastatic cancer [41]. Cancer cell-derived exosomal circ_IARS downregulates ZO-1 levels via inactivating miR-122 in endothelial cells and increases endothelial permeability, eventually reinforcing invasion and metastasis in vivo [41] (Figure 2 and Table 1).

#### 2.5.2. Circ_PDE8A

In various cancer types, miR-338-3p functions as a tumor-suppressive miRNA. By inactivating MAPK signaling, miR-338-3p promotes apoptosis in osteosarcoma cells [99]. In ovarian cancer, the efficacy of cisplatin is augmented by miR-338-3p, which exerts suppressive effects on EMT and cell survival via targeting WNT2B [100]. Similarly, the migration and invasiveness of colorectal cancer cells are repressed by miR-338-3p that targets smoothened (*SMO*) [101]. It was further revealed that miR-338-3p directly controls the expression of metastasis-associated in colon cancer protein 1 (*MACC1*) [47], a transcriptional regulator of MET proto-oncogene (*MET*) [102]. Additional evidence indicated that miR-338-3p activity is limited by circ_PDE8A (also named circ_0036627), which is intensely expressed in pancreatic cancer tissues, plasma exosomes, and cell-derived exosomes [47]. Exosomal circ_PDE8A is capable of being transferred between cancer cells. In addition, this circRNA is able to augment the level of MET via the miR-338-3p/MACC1 axis, stimulating cancer growth and metastasis in vivo [47] (Figure 2 and Table 1).

#### 2.5.3. Circ_ZNF91

Hypoxia-inducible factor-1α (*HIF-1α*) is stabilized by sirtuin 1 (*SIRT1*)-mediated deacetylation. SIRT1 knockdown reduces the invasion ability of cancer cells, together with a decrease in HIF-1α target genes, such as VEGF [103]. SIRT1 can be directly targeted by several miRNAs, including miR-34a-5p and miR-199a, resulting in the attenuation of HIF-1α levels [104,105]. Furthermore, circ_CIDN and circ_0076248 were uncovered to sponge miR-34a-5p and miR-181a, respectively, hence positively regulating SIRT1 expression [106,107].

Likewise, circ_ZNF91 competitively interacts with miR-23b-3p, enhancing HIF-1α stability by upregulating SIRT1 in pancreatic cancer [54]. Circ_ZNF91 is transcriptionally induced by HIF-1α, indicating a positive feedback loop between circ_ZNF91 and HIF-1α. Circ_ZNF91, which is carried in exosomes from hypoxic cancer cells, can be taken up by normoxic cancer cells, leading to gemcitabine resistance and glycolysis. In xenografts, gemcitabine resistance promoted by hypoxic exosomes is reversed by circ_ZNF91 silencing and miR-23b-3p upregulation [54] (Figure 1 and Table 1).

## 3. Exosomal LncRNAs

### 3.1. Colorectal Cancer

#### 3.1.1. CCAL and LINC00659

**Figure 3:**
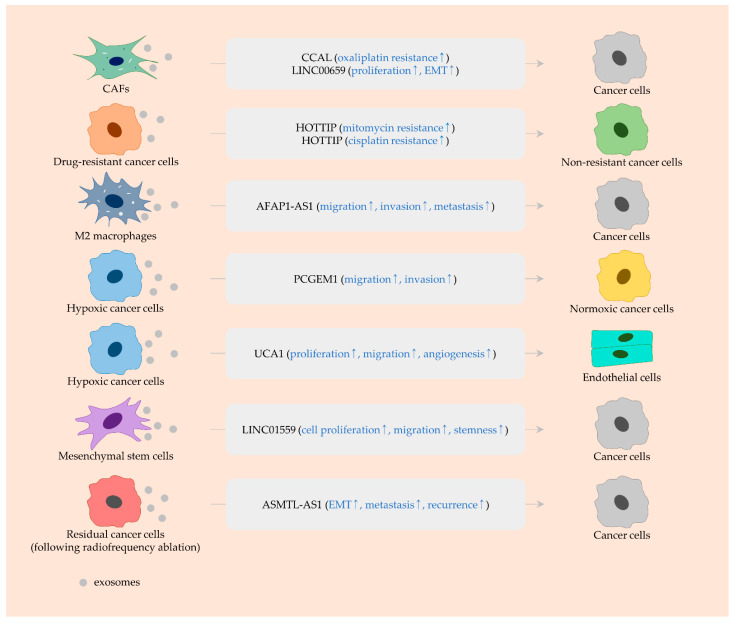
Exosomal lncRNAs derived from various cell types. LncRNAs (black), and their biological functions (blue) are indicated in rounded rectangles. An arrow (↑) indicates the upregulation.

Accumulating evidence has shown that the expression of CCAL is substantially higher in cancer than in non-cancerous tissues and that CCAL promotes angiogenesis and metastasis through escalating, for example, FOXM1 and angiopoietin-like 4 (*ANGPTL4*) expression [73,108]. In addition, CCAL regulates cell proliferation and apoptosis in colorectal cancer and plays a part in 5-FU resistance, owing to its ability to increase ABCB1 levels via activating WNT/β-catenin signaling [109]. Furthermore, CCAL is overexpressed in CAFs and the stromal region in colorectal cancer tissues [110]. Exosomal CCAL released from CAFs is delivered to cancer cells and activates β-catenin via directly interacting with HUR proteins, thus conferring resistance to oxaliplatin [110] (Figure 3 and Table 2).

LncRNA profiling identified that 201 lncRNAs have aberrant levels, including 165 upregulated and 36 downregulated lncRNAs in colorectal cancer. LINC00659 is one of the highly expressed lncRNAs, and the knockdown of this lncRNA suppresses cell cycle progression and cell survival through inactivating PI3K/AKT signaling [111]. Moreover, LINC00659 is abundantly expressed in CAF-derived exosomes, and LINC00659 uptake by cancer cells leads to enhanced proliferation and EMT [112]. A mechanistic investigation proved that LINC00659 acts as a sponge of miR-342-3p, significantly upregulating the expression of annexin A2 (*ANXA2*) [112], which is responsible for the activation of STAT3, an EMT-promoting factor [113] (Figure 3 and Table 2).

**Table 2:** Exosomal lncRNAs in gastrointestinal cancers (in alphanumerical order).

Cargo Molecule	Expression	Source of Exosome	Type of Cancer	Target Molecule	Clinical Relevance	Ref.
AFAP1-AS1	Up	M2 macrophage	Esophageal cancer	miR-26a	-	[114]
ASMTL-AS1	Up	Residual Huh7 cells following heat treatment using a 47 °C water bath	Hepatocellular carcinoma	miR-342-3p	Correlated with distant metastasis and TNM stage	[115]
CCAL	Up	CAFs	Colorectal cancer	HUR	-	[110]
CCAT1	Up	Patient plasma. PANC-1 cells	Pancreatic cancer	miR-138-5p	-	[116]
CEBPA-AS1	Up	BGC-823 and SGC-7901 cells	Gastric cancer	-	Closely associated with Bormann type and TNM stage	[117]
CRNDE-h	Up	Patient serum. SW480, HT29, and LOVO cells	Colorectal cancer	RORγt	Positively associated with the proportion of Th17 cells	[118]
DLX6-AS1	Up	SMMC-7721 and HepG2 cells	Hepatocellular carcinoma	miR-15a-5p	-	[119]
FAM72D-3	Up	Patient serum	Hepatocellular carcinoma	miR-5787	Also upregulated in patients with hepatitis and cirrhosis	[120]
FAM225A	Up	ECA109 and TE-1 cells	Esophageal cancer	miR-206	Associated with advanced stages and poor prognosis	[121]
FMR1-AS1	Up	Serum of female patients. ECA-109 and KYSE-150 cells	Esophageal cancer	TLR7	Associated with a poor clinical outcome of female patients	[122]
FRLnc1	Up	Patient serum. HGC-27 cells	Gastric cancer	-	Associated with TNM stage and lymph node metastasis	[123]
HOTTIP	Up	Mitomycin-resistant SW620 and HCT116 cells	Colorectal cancer	miR-214	Negatively associated with mitomycin response	[124]
	Up	Cisplatin-resistant MGC-803 and MKN-45 cells	Gastric cancer	miR-218	Poor response to cisplatin in patients with high HOTTIP levels	[125]
HULC	Up	Patient serum. PANC-1 cells	Pancreatic cancer	miR-133b	A diagnostic biomarker candidate	[126]
KCNQ1OT1	Up	SW1463 cells	Colorectal cancer	miR-30a-5p	Associated with vascular invasion, lymph node metastasis, and distant metastasis	[127]
LINC00161	Up	Patient serum. Huh7, HCCLM3, MHCC-97L, and MHCC-97H cells	Hepatocellular carcinoma	miR-590-3p	Associated with poor prognosis	[128]
LINC00659	Up	CAFs	Colorectal cancer	miR-342-3p	Highly expressed in patients with poor prognosis	[112]
LINC01133	Up	CFPAC-1 and SW1990 cells	Pancreatic cancer	AXIN2	Associated with poor overall survival and TNM stage	[129]
LINC01559	Up	mesenchymal stem cells	Gastric cancer	miR-1343-3p	Poor prognosis of patients with high LINC01559 levels	[130]
LINC01711	Up	TE-1 cells	Esophageal cancer	miR-326	Associated with poor prognosis	[131]
LINC02418	Up	Patient serum	Colorectal cancer	miR-1273g-3p	Considered as a possible diagnostic marker	[132]
MALAT1	Up	SW620 and LoVo cells	Colorectal cancer	miR-26a and 26b	Worse survival probability of patients with high MALAT1 levels	[133]
PCAT1	Up	Patient serum. Eight cell lines, including KYSE30	Esophageal cancer	miR-326	-	[134]
PCGEM1	Up	Hypoxic AGS and MKN cells	Gastric cancer	SNAI1	-	[135]
POU3F3	Up	KYSE450 and TE12 cells	Esophageal cancer	-	Associated with poor survival rates of patients	[136]
RPPH1	Up	Patient plasma. SW620 and HCT8 cells	Colorectal cancer	TUBB3	Negatively correlated with overall survival	[137]
SPRY4-IT1	Up	Patient serum	Gastric cancer	miR-101-3p	Correlated with tumor size and TNM stage	[138]
TUC339	Up	PLC/PRF/5 cells	Hepatocellular carcinoma	-	-	[139]
UCA1	Up	Patient serum	Colorectal cancer	miR-143	Associated with advanced stages and distant metastasis	[140]
	Up	Patient serum. Hypoxic MIA PaCa-2 cells	Pancreatic cancer	miR-96-5p	Associated with microvascular density, tumor size, lymphatic invasion, TNM stage, and overall survival	[141]
ZFAS1	Up	Eca109 cells	Esophageal cancer	miR-124	Associated with lymph node metastasis, TNM stage, and tumor size	[142]

#### 3.1.2. CRNDE-h, KCNQ1OT1, and RPPH1

**Figure 4:**
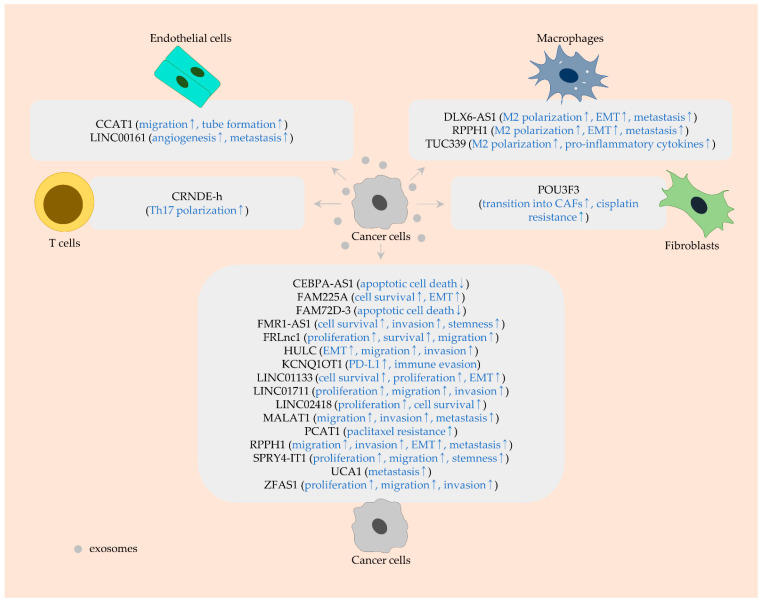
Exosomal lncRNAs derived from cancer cells and their biological functions in endothelial cells, macrophages, T cells, and fibroblasts. Rounded rectangles denote lncRNAs (black) and their functions (blue). Arrows indicate the upregulation (↑) and downregulation (↓).

Cancer-associated lncRNAs affect the tumor immune microenvironment in colorectal cancer. CRNDE-h levels are upregulated in naive CD4+ T cells exposed to cancer cell-secreted exosomes, indicating the transfer of CRNDE-h into CD4+ T cells via exosomes [118]. In addition, it was noted that exosomal CRNDE-h induces the differentiation of peripheral blood mononuclear cells (PBMC) into pro-inflammatory T helper 17 (Th17) cells by binding to and blocking ubiquitin-mediated degradation of RAR-related orphan receptor gammat (RORγt). The knockdown of CRNDE-h reduces the Th17 cell population in company with RORγt downregulation and retards cancer growth in an allograft mouse model [118] (Figure 4 and Table 2).

Numerous studies have suggested that oncogenic KCNQ1OT1 is implicated in migration, invasion, apoptosis, and therapeutic resistance. For example, KCNQ1OT1 significantly increases migration and invasion in hepatocellular carcinomas and osteosarcomas [143,144]. In small cell lung cancer, the inhibition of KCNQ1OT1 induces apoptosis and sensitizes cells to etoposide by interrupting JAK/STAT signaling [145]. Additionally, KCNQ1OT1 expression is upmodulated in methotrexate-resistant colorectal cancer cells, and KCNQ1OT1 silencing re-sensitizes resistant cells to methotrexate through downregulating cAMP-response element binding protein (*CREB*) and CREB-binding protein (*CBP*) levels [146]. Of late, KCNQ1OT1 was observed to support ubiquitin-specific peptidase 22 (*USP22*)-mediated stabilization of PD-L1 by deactivating miR-30a-5p and thereby inhibiting the anti-cancer immunity of CD8+ T cells [127]. Since KCNQ1OT1 is secreted from cancer cells via exosomes, the reciprocal transfer of KCNQ1OT1 between cancer cells can substantially increase PD-L1 levels, contributing to immune evasion [127] (Figure 4 and Table 2). KCNQ1OT1 knockdown may activate other oncogenic factors, since miR-30a-5p is intriguingly capable of negatively regulating tumor-suppressive genes [147,148].

Tubulin beta 3 class III (*TUBB3*) is induced by SNAI1 and facilitates cell migration and invasion [149]. The activity of focal adhesion kinase (*FAK*), SRC proto-oncogene (*SRC*), and STAT3 is downregulated by TUBB3 silencing, thus restraining metastasis [150]. Furthermore, it was proven that RPPH1 physically binds to and stabilizes TUBB3 proteins in colorectal cancer cells [137]. Since cancer cell-derived exosomes contain RPPH1, this lncRNA is able to affect TUBB3 expression in other surrounding cancer cells, encouraging colorectal cancer progression. Moreover, exosomal RPPH1 can mediate M2 polarization of macrophages, ultimately promoting migration, invasion, EMT, and metastasis [137] (Figure 4 and Table 2).

#### 3.1.3. HOTTIP

Several studies have indicated that HOTTIP is interconnected with drug resistance. HOTTIP desensitizes lung cancer cells to doxorubicin, etoposide, and cisplatin, through increasing BCL2 levels [151]. Downregulation of HOTTIP turns WNT/β-catenin signaling inactive, improving anti-prostate cancer effects of cisplatin [152]. Similarly, HOTTIP enhances stemness by attenuating miR-205 effects and confers resistance to cisplatin in ovarian cancer [153].

In colorectal cancer, HOTTIP is overexpressed in mitomycin-resistant cancer cells and their exosomes. Exosomes from resistant cells weaken mitomycin-induced DNA damage in non-resistant cells via conveying HOTTIP, which impairs miR-214-mediated inhibition of karyopherin subunit alpha 3 (*KPNA3*) expression [124] (Figure 3 and Table 2). As mentioned above, HOTTIP inactivates miR-205. In endothelial cells, miR-205 can activate AKT signaling, thus promoting angiogenesis [154]. Therefore, careful monitoring of the effect of exosomal HOTTIP on angiogenesis is demanded.

#### 3.1.4. LINC02418

A few studies provided evidence that LINC02418 is an oncogenic lncRNA. LINC02418 positively regulates lung cancer cell proliferation while limiting apoptotic cell death by sponging miR-4677-3p [155,156]. In a similar fashion, LINC02418 constrains apoptosis via controlling the miR-34b-5p/BCL2 axis, promoting colorectal cancer progression [157]. In addition, LINC02418 is overexpressed in colorectal cancer tissues, cell lines, and serum exosomes, suggesting the likelihood of the origin of exosomal LINC02418 from cancer cells [132]. Furthermore, it was discerned that LINC02418 upregulates the level of MELK by disabling miR-1273g-3p to support proliferation and cell survival in cancer cells [132]. These findings demonstrate that exosomal LINC02418 from cancer cells can act as one of the bioactive molecules in recipient cancer cells (Figure 4 and Table 2). However, it should be noted that miR-1273g-3p has a stimulatory effect on cell proliferation, migration, and invasion in LoVo cells, a colorectal cancer cell line [158]. Such findings indicate the interaction of miR-1273g-3p with both oncogenes and tumor-suppressive genes, eliciting different functions depending on the cellular context.

#### 3.1.5. MALAT1 and UCA1

Exosomal MALAT1 and UCA1 serve as pro-metastatic factors in colorectal cancer [133,140]. Exosomal MALAT1 from metastatic cells can be internalized into other cancer cells and raise their migratory and invasive capacity in vitro. Furthermore, liver and lung metastases of colorectal cancer are advanced by exosomal MALAT1 in vivo. Such pro-metastatic effect of MALAT1 is partly due to the activation of PI3K/AKT signaling via sponging miR-26a and miR-26b [133] (Figure 4 and Table 2).

UCA1 is highly expressed in colorectal cancer tissues, cell lines, and patients’ serum exosomes [140]. This suggests the possibility that exosomal UCA1 is derived from cancer cells. Further, it was found that cancer cells can gain metastatic potential following treatment with serum exosomes harboring UCA1. In this study, it was also proposed that UCA1 is capable of impelling metastasis by downmodulating and upmodulating miR-143 and myosin VI (*MYO6*) levels, respectively [140] (Figure 4 and Table 2). However, UCA1 exerts anti-migratory and -invasive roles in esophageal cancer [159], showing that the function of UCA1 is dissimilar, depending on cancer types.

### 3.2. Esophageal Cancer

#### 3.2.1. AFAP1-AS1

AFAP1-AS1, a highly expressed lncRNA in multiple cancers, was discovered to promote cancer progression, stemness, as well as therapeutic resistance. AFAP1-AS1 possesses stimulatory effects on cell proliferation, migration, and invasion in thyroid cancer as a consequence of sequestering miR-204-3p [160]. In laryngeal cancer, AFAP1-AS1 potentiates stemness and cisplatin resistance by raising the level of recombination signal-binding protein for immunoglobulin Kappa J region (*RBPJ*) [161].

Besides this, a profuse amount of AFAP1-AS1 was detected in exosomes unleashed by M2 macrophages. Exosomal AFAP1-AS1 can enter esophageal cancer cells and interact with miR-26a to promote the level of cyclic AMP-responsive element-binding protein 2 (*CREB2*, also known as activating transcription factor 2 (*ATF2*)), thereby boosting migration/invasion in vitro and lung metastasis in vivo [114] (Figure 3 and Table 2).

#### 3.2.2. FAM225A and FMR1-AS1

Neuropilin and tolloid-like 2 (*NETO2*) activates the PI3K/AKT signaling-mediated NF-κB/SNAI1 axis, triggering the EMT process [162]. The silencing of NETO2 can induce apoptosis, along with an increase in cleaved caspase-3 and a decrease in BCL2 [163]. In addition, forkhead box P1 (*FOXP1*) supports cell survival via transcriptionally repressing the expression of proapoptotic genes, such as BCL2 interacting killer (*BIK*) [164]. Furthermore, the level of EMT-related genes, including SNAI1, is positively affected by FOXP1 overexpression [165]. Both NETO2 and FOXP1 are targets of miR-206 and are upregulated by FAM225A in esophageal cancer. FOXP1 can increase FAM225A levels, indicating a positive feedback loop between these molecules [121]. FAM225A is located in exosomes from cancer cells. Therefore, exosomal FAM225A can be transferred to other cancer cells, inciting cell survival and EMT [121] (Figure 4 and Table 2).

FMR1-AS1 is a highly expressed lncRNA and is sorted into exosomes in esophageal cancer [122]. This exosomal lncRNA is transcriptionally induced by NF-κB, which, in turn, activates NF-κB-MYC signaling by binding to toll-like receptor 7 (*TLR7*). Thus, FMR1-AS1 exerts anti-apoptotic and pro-invasive effects and strengthens stemness in recipient cancer cells. In vivo observation also indicated that exosomal FMR1-AS1 fuels cancer growth [122] (Figure 4 and Table 2).

#### 3.2.3. LINC01711 and ZFAS1

LINC01711 is upregulated in esophageal cancer tissues and several cell lines. In addition, this lncRNA can be contained in exosomes produced by cancer cells, suggesting that exosomal LINC01711 can play an oncogenic role [131]. It has been shown that exosomal LINC01711 dampens the activity of miR-326, which targets fascin actin-bundling protein 1 (*FSCN1*), in recipient cancer cells, hence expediting proliferation, migration, and invasion [131] (Figure 4 and Table 2). Another study showed the positive correlation between LINC01711 and TGFB1 expression [166]. Thus, miR-326 activity may be modulated by the TGFB1/LINC01711 axis. FSCN1 is markedly increased in diverse cancers and affects various cellular processes, such as invasion, EMT, metastasis, as well as drug resistance [167,168,169]. Therefore, it is worth considering LINC01711 as a prospective target for esophageal cancer treatment.

In esophageal cancer, exosomal ZFAS1 is taken up by other surrounding cancer cells and exerts a positive influence on proliferation, migration, and invasion via the miR-124/STAT3 axis [142] (Figure 4 and Table 2). ZFAS1 has been known to abrogate tumor-suppressive functions of numerous miRNAs in gastrointestinal cancers. By targeting miR-150, ZFAS1 upregulates MMP14, MMP16, and zinc finger E-box-binding homeobox 1 (*ZEB1*) expression, consequently increasing the occurrence of lung metastasis in hepatocellular carcinomas [170]. ZFAS1 also invalidates miR-150 activity and facilitates angiogenesis in colorectal cancer. Of note, ZFAS1 enhances tube formation of HUVECs [171]. Exosomal ZFAS1 may therefore contribute to angiogenesis by affecting the biological traits of nearby endothelial cells in esophageal cancer.

#### 3.2.4. PCAT1 and POU3F3

PCAT1 and POU3F3 have been regarded as oncogenic lncRNAs. PCAT1 physically binds to enhancer of Zeste 2 polycomb repressive complex 2 subunit (*EZH2*) and, thus, epigenetically silences PTEN expression, leading to cisplatin resistance in gastric cancer [172]. In hepatocellular carcinomas, PCAT1 silencing downregulates the level of high mobility group box 1 (*HMGB1*) and incapacitates the invasive and migratory potential [173]. In a similar manner, the degree of apoptosis and EMT process is attenuated in POU3F3-silenced colorectal cancer cells [174].

In esophageal cancer, both PCAT1 and POU3F3 regulate drug sensitivity [134,136]. PCAT1 was validated to sponge miR-326, which has an inhibitory action on AKT. Downregulation of PCAT1 induces G2/M cell cycle arrest and turns cancer cells more sensitive to paclitaxel [134]. This lncRNA is present in exosomes from cancer cells [134], demonstrating its potential to cause paclitaxel resistance in recipient cancer cells (Figure 4 and Table 2).

POU3F3 is also encompassed by exosomes generated from cancer cells and delivered to fibroblasts. POU3F3 ultimately accelerates the conversion of normal fibroblasts (NF) into CAFs, although the mechanisms of POU3F3-mediated fibroblast activation remain unclear. CAFs are then responsible for cisplatin resistance by secreting IL6 [136] (Figure 4 and Table 2). POU3F3 has been shown to upregulate the expression of rho-associated coiled-coil containing protein kinase (*ROCK*) [175]. ROCK is known to activate YAP1 [176], which stimulates NF-CAF transition [177]. For this reason, POU3F3 may regulate fibroblast activation via the ROCK/YAP1 axis.

### 3.3. Gastric Cancer

#### 3.3.1. CEBPA-AS1

CEBPA-AS1 deactivates Notch signaling via sponging of oncogenic miR-10b-5p, and it increases apoptotic cell death in osteosarcomas [178]. By contrast, CEBPA-AS1 is highly expressed in exosomes from gastric cancer cells and suppresses apoptotic cell death in recipient cancer cells [117] (Figure 4 and Table 2). CEBPA-AS1 partakes in BCL2 upregulation in oral squamous cell carcinomas [179]. Although it is required to unravel the mechanisms underlying the oncogenic role of CEBPA-AS1 in gastric cancer, apoptosis may nonetheless be regulated by the CEBPA-AS1/BCL2 axis in gastric cancer as well.

#### 3.3.2. FRLnc1 and PCGEM1

Exosomal FRLnc1 from gastric cancer cells can effectively augment cell proliferation, viability, and migration in receiver cancer cells [123] (Figure 4 and Table 2). Although direct targets of FRLnc1 remain to be identified, FRLnc1 is known to upregulate the level of TGFB1 and TWIST, favoring migration in vitro and lung metastasis in vivo in gastric cancer [180]. In addition, FRLnc1 can be induced by FOXM1 [180], and CCAL is capable of upmodulating FOXM1 levels (Section 3.1.1). Thus, CCAL may increase FRLnc1 expression in gastric cancer, resulting in an abundance of FRLnc1 in exosomes.

PCGEM1 stabilizes SNAI1 proteins via physically interacting with them, serving as a pro-migratory and -invasive lncRNA in gastric cancer [135]. Hypoxic cancer cells are sources of plentiful amounts of exosomal PCGEM1, which can be internalized into normoxic cancel cells. Exosomal PCGEM1 leads to an increase in SNAI1 levels and expedites migration and invasion in normoxic cancer cells [135] (Figure 3 and Table 2). Evidence from another study suggested that PCGEM1 also improves the invasive ability of gastric cancer cells by increasing the level of prolyl 4-hydroxylase subunit alpha 2 (*P4HA2*) [181]. Accordingly, PCGEM1 may transcriptionally and post-transcriptionally regulate SNAI1 expression, since P4HA2 transcriptionally induces SNAI1 [182].

#### 3.3.3. HOTTIP

As mentioned in Section 3.1.3, HOTTIP regulates cellular factors and signaling linked to therapeutic resistance. In gastric cancer, cisplatin resistance in recipient cells is instigated by exosomal HOTTIP secreted from cisplatin-resistant cells. Mechanistically, exosomal HOTTIP augments the expression of high mobility group AT-hook 1 (*HMGA1*) via nullifying miR-218 activity [125] (Figure 3 and Table 2). In addition to cisplatin, HMGA1 contributes to cellular resistance to paclitaxel, 5-FU, and gefitinib [183,184,185]. Therefore, targeting HOTTIP may beneficially improve the effectiveness of various kinds of anti-cancer drugs.

#### 3.3.4. LINC01559

Research-based evidence demonstrated the role of LINC01559 as an oncogenic lncRNA in gastrointestinal cancers. In pancreatic cancer, LINC01559 interacts with and enhances the transcriptional activity of YAP, boosting cell proliferation and migration [186]. Through sponging miR-6783-3p, LINC01559 renders hepatocellular carcinoma insensitive to oxaliplatin [187]. In addition, LINC01559 stabilizes ZEB1 mRNA via interacting with IGF2BP2 and acts as an EMT-promoting lncRNA in gastric cancer. ZEB1, in turn, transcriptionally activates LINC01559 expression, indicating a LINC01559–ZEB1 feedback loop [188].

Moreover, it was noticed that exosomes from mesenchymal stem cells convey LINC01559 to gastric cancer cells [130]. Exosomal LINC01559 counteracts the negative effect of miR-1343-3p on phosphoglycerate kinase 1 (*PGK1*) and epigenetically inhibits PTEN expression. By regulating PGK1 and PTEN levels, exosomal LINC01559 can activate PI3K/AKT signaling and promote cell proliferation, migration, and stemness in gastric cancer cells [130] (Figure 3 and Table 2).

#### 3.3.5. SPRY4-IT1

SPRY4-IT1 has been proven to serve as either a tumor-suppressive or an oncogenic lncRNA. SPRY4-IT1 is downregulated in cisplatin-resistant lung cancer cells and takes part in the repression of EMT. Overexpression of SPRY4-IT1 reinforces cisplatin-induced apoptosis in resistant cells [189]. Conversely, EMT can be triggered by SPRY4-IT1 in cervical cancer, colorectal cancer, and hepatocellular carcinomas [190,191,192]. What is more, SPRY4-IT1 is transcriptionally activated by NF-κB and promotes staufen-mediated degradation of transcription elongation factor B subunit 1 (*TCEB1*) mRNA to upregulate HIF-1α levels [193]. Thus, it is probable that SPRY4-IT1 can control the expression of other ncRNAs, such as circ_ZNF91, which is regulated by HIF-1α (see Section 2.5.3 about circ_ZNF91).

Furthermore, SPRY4-IT1 is enriched in gastric cancer tissues, cancer cell lines, and serum exosomes [138]. Hence, exosomal SPRY4-IT1 can be released from cancer cells and regulate the biological properties of other cancer cells. SPRY4-IT1 functionally provokes cell proliferation, migration, and stemness via regulating the miR-101-3p/AMP-activated protein kinase (*AMPK*) axis [138] (Figure 4 and Table 2).

### 3.4. Hepatocellular Carcinoma

#### 3.4.1. ASMTL-AS1

ASMTL-AS1 has a dual role in cancer. Overexpression of ASMTL-AS1 stabilizes spermidine/spermine N1-acetyltransferase 1 (*SAT1*) mRNA, retarding the growth of lung cancer along with ferroptosis [194]. ASMTL-AS1 impairs WNT/β-catenin signaling, restraining breast cancer growth in vivo [195]. By contrast, ASMTL-AS1 exacerbates osteosarcoma progression through the upmodulation of ADAM metallopeptidase domain 9 (*ADAM9*) expression [196].

In hepatocellular carcinoma, ASMTL-AS1 is upregulated in cancer tissues and further increased in residual tissues following insufficient radiofrequency ablation (RFA) [115]. Such an expression pattern of ASMTL-AS1 implies that this lncRNA may contribute to cancer recurrence. Indeed, ASMTL-AS1 is transcriptionally elevated by MYC and increases Nemo-like kinase (*NLK*) expression via rendering miR-342-3p ineffective, fundamentally reinforcing malignant phenotypes, such as EMT and metastasis [115]. Compared to its levels in non-treated cells, ASMTL-AS1 levels are increased in residual cancer cells following heat treatment mimicking insufficient RFA. In addition, ASMTL-AS1 can be enclosed by exosomes and conveyed from residual cells into other cancer cells [115]. These observations demonstrate the contribution of exosomal ASMTL-AS1 to cancer recurrence by increasing NLK signaling (Figure 3 and Table 2).

#### 3.4.2. DLX6-AS1 and TUC339

Exosomal lncRNAs from hepatocellular carcinoma cells are accountable for M2 polarization, following an uptake by macrophages. Coculturing cancer cells with M2 macrophages stimulated by exosomal DLX6-AS1 promotes migration, invasion, and EMT in vitro. Furthermore, by activating M2 polarization, exosomal DLX6-AS1 triggers lung metastasis in vivo. Exosomal DLX6-AS1 exerts its function by upregulating C-X-C motif chemokine ligand 17 (*CXCL17*), which is targeted by miR-15a-5p [119] (Figure 4 and Table 2). Despite DLX6-AS1’s oncogenic ability, the knockdown of this lncRNA activates autophagy [197]. Since autophagy can be considered as the pro-survival pathway [198], double inhibition of DLX6-AS1 and autophagy may efficiently suppress cancer and synergistically improve the anti-cancer effect of conventional treatment.

In the case of TUC339, more studies are needed to unveil how TUC339 controls M2 polarization and what miRNAs and proteins this lncRNA interacts with once transferred to macrophages. Nonetheless, it was observed that exosomal TUC339 from cancer cells is required for M2 polarization and an increase in pro-inflammatory cytokines [139]. These results suggest that the downregulation of exosomal TUC339 can conceivably control cancer progression (Figure 4 and Table 2).

#### 3.4.3. FAM72D-3

FAM72D-3 is one of the upregulated lncRNAs in serum exosomes from patients with hepatocellular carcinomas. FAM72D-3 silencing increases apoptotic cell death [120]. Thus, it is conceivable that the transfer of cancer cell-derived exosomal FAM72D-3 to other cancer cells enables them to escape apoptosis. In addition, miR-5787 levels are elevated in FAM72D-3-silencing cells, suggesting miR-5787 as a possible downstream effector of FAM72D-3 [120]. It is important to define whether FAM72D-3 directly regulates miR-5787 or not and if miR-5787 is an oncogenic or a tumor-suppressive factor (Figure 4 and Table 2).

#### 3.4.4. LINC00161

LINC00161 upregulates interferon-induced protein with tetratricopeptide repeats 2 (*IFIT2*), a cell death-promoting factor, augmenting cisplatin-induced apoptosis in osteosarcoma cells [199]. On the other hand, LINC00161 is one of the highly expressed lncRNAs in cisplatin-resistant ovarian cancer cells and causes cisplatin resistance by upregulating ERK [200]. Besides, intercellular transport of LINC00161 from cancer cells to endothelial cells was noticed in hepatocellular carcinomas [128]. Exosomal LINC00161 can increase the level of ROCK by binding to miR-590-3p in endothelial cells, facilitating proliferation, migration, and tube formation. The silencing of LINC00161 markedly inhibits angiogenesis and metastasis in vivo [128] (Figure 4 and Table 2).

### 3.5. Pancreatic Cancer

#### 3.5.1. CCAT1

In addition to the resistance-associated role of HMGA1 (Section 3.3.3), HMGA1 is involved in angiogenesis. For instance, HMGA1 can increase the transcriptional activity of FOXM1 on angiogenesis-related genes, such as VEGF [201]. In addition, HMGA1 is able to interact with HIF-1, thus mediating a hypoxia-induced increase in VEGF [202]. A recent report demonstrated that CCAT1 is released from pancreatic cancer cells through exosomes and positively modulates the level of HMGA1, a miR-138-5p target, in endothelial cells [116]. The effect of CCAT1 on migration and tube formation of endothelial cells is antagonized by miR-138-5p overexpression [116]. These results suggest that exosomal CCAT1 may regulate autocrine VEGF signaling via the miR-138-5p/HMGA1/VEGF axis in endothelial cells (Figure 4 and Table 2).

#### 3.5.2. HULC

Identification of lncRNAs regulated by TGFB1, an EMT-promoting factor, showed that HULC is among the TGFB1-induced lncRNAs in pancreatic cancer cells. Both intracellular and intra-exosomal levels of HULC are upregulated by TGFB1 [126]. Treatment with exosomes derived from donor cancer cells increases the expression of HULC and EMT markers, such as vimentin, in recipient cancer cells and promotes invasion and migration. These tendencies are further reinforced by the treatment with exosomes derived from TGFB1-treated cancer cells. In addition, it was found that the effect of HULC is due to its suppressive action on miR-133b, an EMT-inhibiting miRNA [126] (Figure 4 and Table 2).

#### 3.5.3. LINC01133

Disparate functions of LINC01133 depending on cancer types have been reported. LINC01133 hampers gastric and bladder cancer progression via somatostatin upregulation and WNT signaling inactivation, respectively [203,204]. Consistently, by suppressing SRY-box transcription factor 4 (*SOX4*) expression, LINC01133 is capable of inhibiting metastasis in breast cancer [205]. On the contrary, LINC01133 facilitates EMT via enhancing SNAI1 levels and activates STAT3 signaling in hepatocellular carcinoma [206]. In ovarian cancer, LINC01133 upregulates tumor protein D52 (*TPD52*) levels through sharing the same binding sites with miR-495-3p, acting as a pro-metastatic factor [207]. It was also identified that LINC01133 is transactivated by CCAAT/enhancer-binding protein β (*CEBPB*) and positively modulates cell proliferation via activating cyclin G1 expression in pancreatic cancer [208].

Moreover, LINC01133 is highly expressed in pancreatic cancer cells as well as in their secreted exosomes. LINC01133 interacts with EZH2 to silence the expression of axis inhibition protein 2 (*AXIN2*), activating β-catenin and promoting cell survival, proliferation, and EMT [129]. In this study, periostin (*POSTN*) was discovered to increase LINC01133 via the epidermal growth factor receptor (*EGFR*)/MYC axis and promote exosome secretion in pancreatic cancer cells [129]. These findings suggest that exosomal LINC01133 augmented by POSTN in donor cells can affect the progression of recipient cells via the AXIN2/β-catenin axis (Figure 4 and Table 2).

#### 3.5.4. UCA1

Angiomotin-like 2 (*AMOTL2*) activates the SRC/ERK signaling pathway, playing a critical role in migration and proliferation of endothelial cells during angiogenesis [209]. More recently, exosomal UCA1 was proven to be released from hypoxic pancreatic cancer cells and transported to endothelial cells, in which UCA1 relieves the activity of miR-96-5p on AMOTL2. Exosomal UCA1 secreted from hypoxic cancer cells reinforces cancer growth and angiogenesis in vivo [141] (Figure 3 and Table 2).

## 4. Conclusions

Growing evidence introduced here shows that exosomal circRNAs and lncRNAs are involved in the intercellular communication between a wide range of cell types. Knockdown of a single exosomal ncRNA sufficiently blocks cancer progression and aggressiveness; therefore, targeting ncRNAs can be a reasonable option for gastrointestinal cancer treatment.

Nonetheless, it is critical to consider the characteristic features of these ncRNAs. These include the differences in function according to the type of cancer and cell. Inhibition of appropriately selected ncRNAs that are specifically relevant to the cancer type is required. Furthermore, to avoid side effects on normal cells, cancer-specific suppression of oncogenic ncRNA is essential. Additionally, even targeting ncRNAs themselves may hardly be free from therapeutic resistance. Therefore, it is attractive to explore innovative combination therapy with ncRNA inhibition and other anti-cancer treatments for pronouncedly controlling resistance problems.

In addition to the inhibition of exosome cargo, restraint of exosome secretion has therapeutic benefits. However, pharmacological agents, such as GW4869, are non-specific inhibitors of exosome-mediated intercellular dialogues and have undesirable effects [210,211]. Besides this, it has been found that exosome uptake can be controlled by cellular factors such as advanced glycosylation end-product specific receptor (*AGER*) [212]. Therefore, identifying genes involved in the secretion and uptake of exosomes will open the way for more specific inhibition of the cancer-supporting activity of exosomes through gene knockdown approaches.

Overall, an advanced understanding of the nature of ncRNAs and the mechanism underpinning the exosome itinerary will pave the way for overcoming gastrointestinal cancer.

## Data Availability

Not applicable.
